# Supplementary material for: REM disruption and REM vagal activity predict extinction recall in trauma-exposed individuals
Source: Psychol Med. 2024 Dec 9;54(16):4623–34. doi: 10.1017/S0033291724002757 (PMC11769908; doi:10.1017/S0033291724002757)
Supplement: Yuksel et al. supplementary material 1 — Yuksel et al. supplementary material [file S0033291724002757sup001.pdf]

SUPPLEMENTARY MATERIALS

Participant Enrollment

One hundred and ninety-five participants were enrolled of whom 132 completed the study. Thirty-seven participants were excluded because exclusion criteria were detected during the clinical interview. Twenty-one participants withdrew consent: Nineteen participants left the study due to scheduling conflicts, 1 participant met exclusion criteria while undergoing the study, and 1 dropped out due to medical issues unrelated to the study. Finally, the participation of 5 subjects was terminated by the investigators, because they could not tolerate the MRI environment (n = 4) and polysomnography (n = 1). Participants were compensated $500 if they completed the study.

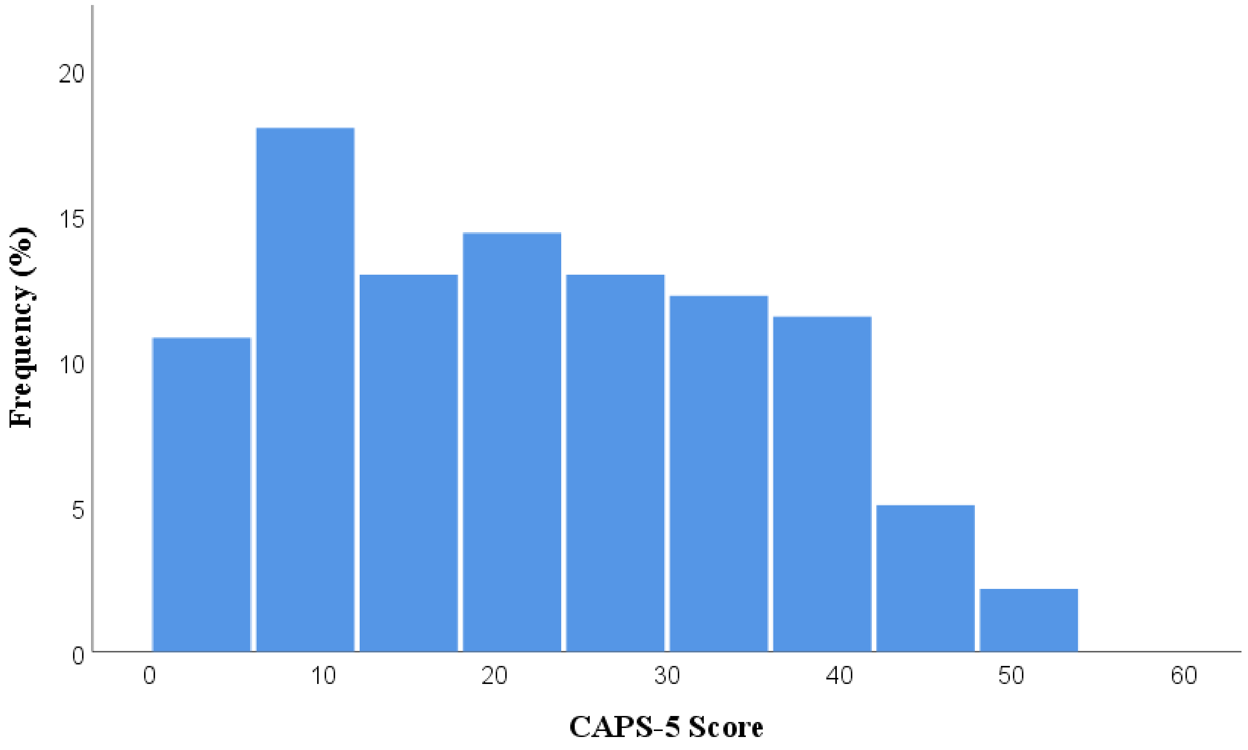

Figure S1. Distribution of CAPS-5 score in the sample.

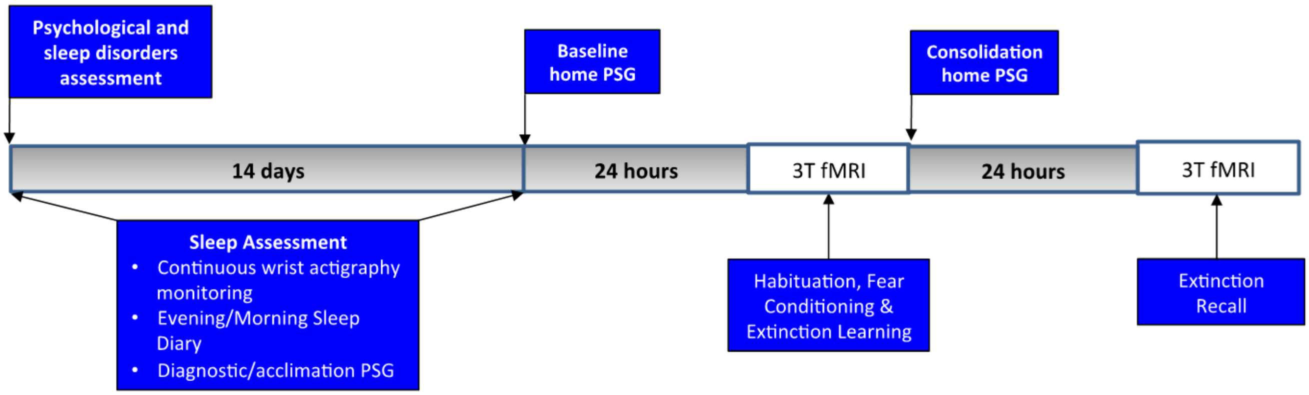

Figure S2. Timeline of the study procedures. The sleep data analyzed in this study was obtained during the consolidation night.

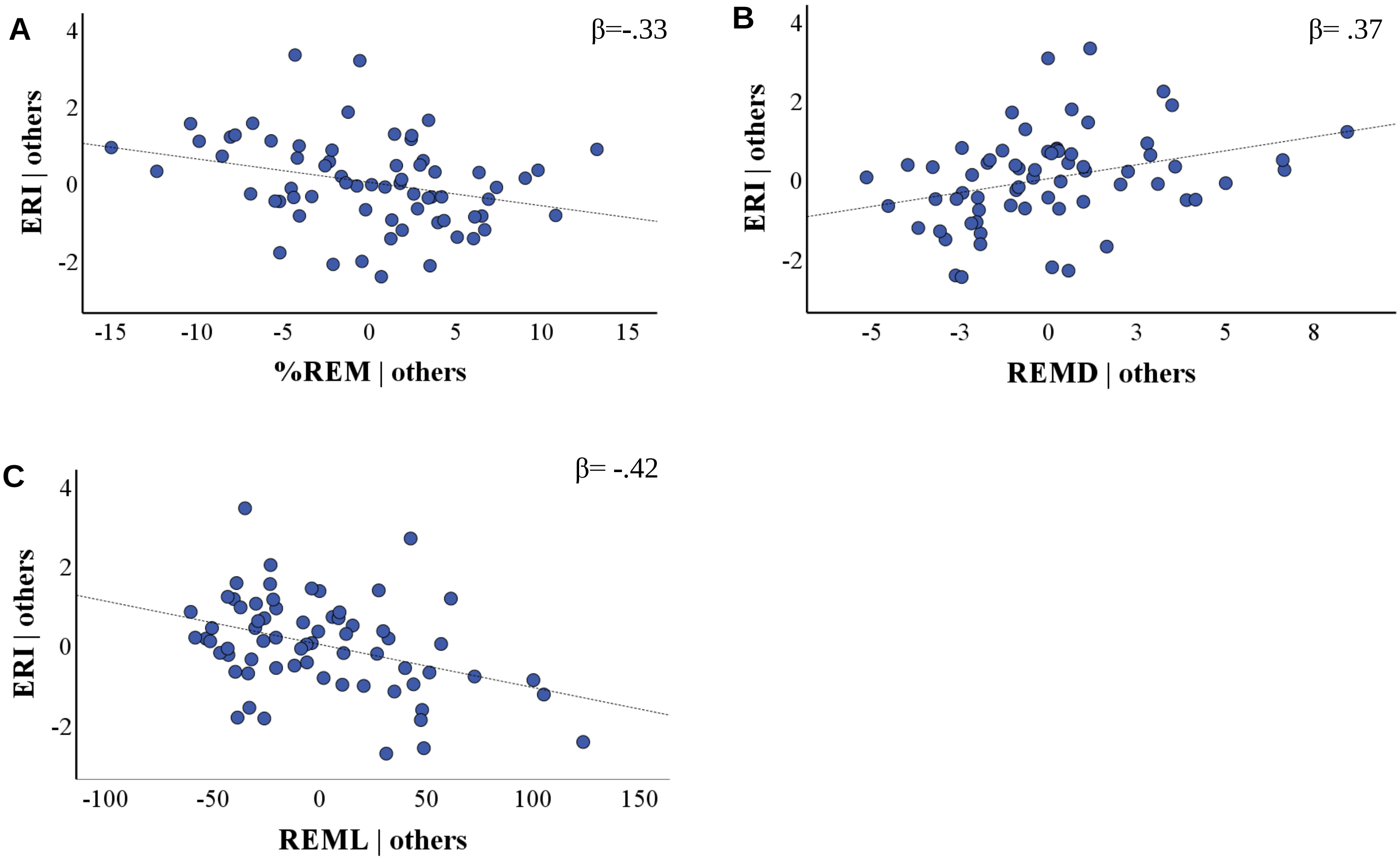

Figure S3. Partial regression (added variable) plots of the significant REM variables in the regression analyses that tested the hypothesis 1 for physiological extinction recall (ERI). The Y axis represents the residuals derived from regressing ERI on all the predictor variables in the corresponding model, except the variable noted on the X axis. The X axis represents the residuals derived from regressing the predictor variable noted on the X axis on all the other predictor variables in the corresponding models. The slope reflects the standardized partial regression coefficient (β). Note that smaller ERI denotes better physiological extinction recall. HF[ms^2]: Absolute power of high frequency heart rate variability; %REM: Proportion of REM sleep to total sleep time; REMD: REM density; REML: REM latency.

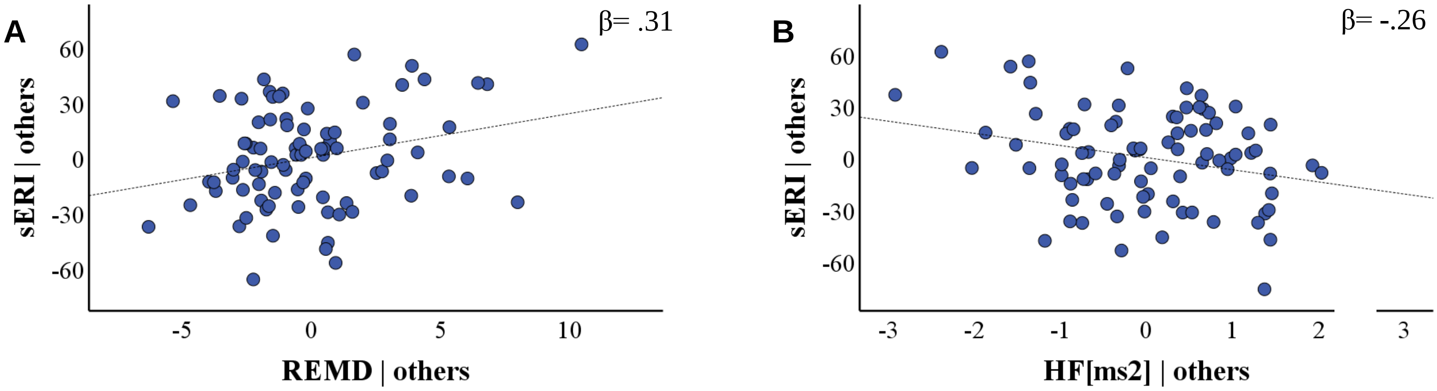

Figure S4. Partial regression (added variable) plots of the significant REM variables in the regression analyses that tested the hypothesis 1 (A), and the hypothesis 2 (B), for subjective extinction recall (sERI). The Y axis represents the residuals derived from regressing sERI on all the predictor variables in the corresponding model, except the variable noted on the X axis. The X axis represents the residuals derived from regressing the predictor variable noted on the X axis on all the other predictor variables in the corresponding models. The slope reflects the standardized partial regression coefficient (β). Note that smaller sERI denotes better subjective extinction recall. HF[ms^2]: Absolute power of high frequency heart rate variability; REMD: REM density.

Model	Predictors	B	SE	β	t	p	95% CI						
1	**Sex**	-.603	.351	-.242	-1.714	.092	-1.307	.102					
	**Medication**	-.075	.396	-.025	-.189	.850	-.869	.719					
	**%REM**	-.052	.026	-.281	-2.004	.050	-.104	.000					
	**REMD**	.147	.053	.391	2.793	.007	.041	.253					
	**REML**	-.010	.004	-.383	-2.688	.010	-.017	-.003					
	**REMF**	.034	.032	.148	1.035	.305	-.032	.099					
									**Change**			**ANOVA**	
									**ΔF**	**p**	**Adj. R^2**	**F**	**p**
2	**Sex**	-.651	.345	-.261	-1.886	.065	-1.344	.042	2.553	.088	.162	2.450	**.025**
	**Medication**	.064	.396	.022	.162	.872	-.730	.859					
	**%REM**	-.058	.026	-.313	-2.280	**.027**	-.109	-.007					
	**REMD**	.159	.053	.423	3.030	**.004**	.054	.265					
	**REML**	-.012	.004	-.446	-3.122	**.003**	-.019	-.004					
	**REMF**	.028	.032	.124	.885	.380	-.036	.092					
	**HF[ms^2]**	-.401	.201	-.345	-1.999	.051	-.803	.002					
	**HF[ms^2]×Sex**	.635	.294	.391	2.160	**.035**	.045	1.224					

Table S1. Hierarchical regression analysis for physiological extinction recall (ERI) with all REM variables included in the model. Note that smaller ERI denotes better extinction recall. HF[ms^2]: Absolute power of high frequency heart rate variability; %REM: Proportion of REM sleep to total sleep time; REMD: REM density; REMF: REM fragmentation; REML: REM latency.

Model	Predictors	B	SE	β	t	p	95% CI						
1	Age	-.265	.655	-.045	-.404	.687	-1.569	1.039					
	Sex	.029	7.341	.000	.004	.997	-14.589	14.646					
	Medication	-17.151	8.405	-.232	-2.041	.045	-33.886	-.415					
	%REM	.246	.543	.055	.453	.652	-.836	1.328					
	REMD	2.663	.993	.317	2.681	.009	.685	4.640					
	REML	.066	.078	.103	.847	.400	-.090	.222					
	REMF	.823	.671	.145	1.227	.224	-.513	2.159					
									Change			ANOVA	
									ΔF	p	Adj. R^2	F	p
2	Age	-.433	.640	-.073	-.676	.501	-1.707	.842					
	Sex	1.142	7.143	.018	.160	.873	-13.083	15.368	5.681	**.020**	.137	2.671	**.012**
	Medication	-20.916	8.312	-.283	-2.516	**.014**	-37.471	-4.362					
	%REM	.217	.528	.048	.411	.682	-.834	1.268					
	REMD	2.399	.971	.286	2.472	**.016**	.466	4.332					
	REML	.067	.076	.103	.877	.383	-.085	.218					
	REMF	.800	.651	.141	1.228	.223	-.498	2.097					
	HF[ms^2]	-6.940	2.912	-.251	-2.384	**.020**	-12.740	-1.141					

Table S2. Hierarchical regression analysis for subjective extinction recall (sERI) with all REM variables included in the model. Note that smaller sERI denotes better extinction recall. HF[ms^2]: Absolute power of high frequency heart rate variability; %REM: Proportion of REM sleep to total sleep time; REMD: REM density; REMF: REM fragmentation; REML: REM latency.

Model	Predictors	B	SE	β	t	p	95% CI						
1	**Sex**	-.531	.345	-.213	-1.539	.130	-1.221	.160					
	**Medication**	.017	.386	.006	.045	.964	-.757	.791					
	**%REM**	-.043	.025	-.234	-1.763	.083	-.093	.006					
	**REMD**	.143	.053	.379	2.718	.009	.037	.248					
	**REML**	-.008	.003	-.323	-2.481	.016	-.015	-.002					
									**Change**		**ANOVA**		
									**ΔF**	**p**	**Adj. R^2**	**F**	**p**
2	**Sex**	-.581	.337	-.233	-1.726	.090	-1.256	.094	2.700	0.076	0.164	2.686	**0.019**
	**Medication**	.088	.382	.030	.231	.818	-.679	.855					
	**%REM**	-.049	.024	-.266	-2.051	**.045**	-.098	-.001					
	**REMD**	.151	.052	.402	2.914	**.005**	.047	.256					
	**REML**	-.010	.003	-.390	-2.981	**.004**	-.017	-.003					
	**RMSSD**	-.872	.396	-.375	-2.200	**.032**	-1.667	-.077					
	**RMSSD×Sex**	1.209	.580	.369	2.085	**.042**	.046	2.371					

Table S3. Hierarchical regression analysis for physiological extinction recall (ERI). RMSSD and RMSSD × Sex interaction were significant predictors. Note that smaller ERI denotes better extinction recall. %REM: Proportion of REM sleep to total sleep time; REMD: REM density; REML: REM latency; RMSSD: Root mean square of successive differences.

Model	Predictors	B	SE	β	t	p	95% CI						
**1**	**Age**	-.506	.630	-.085	-.803	.424	-1.761	.748					
	**Sex**	-.224	7.153	-.004	-.031	.975	-14.458	14.010					
	**Medication**	-14.982	8.278	-.203	-1.810	.074	-31.456	1.492					
	**REMD**	2.917	.970	.348	3.006	.004	.986	4.849					
									**Change**		**ANOVA**		
									**ΔF**	**p**	**Adj. R^2**	**F**	**p**
**2**	**Age**	-.628	.620	-.106	-1.013	.314	-1.861	.605	4.515	**0.037**	0.133	3.265	**0.010**
	**Sex**	.062	7.002	.001	.009	.993	-13.875	13.999					
	**Medication**	-18.339	8.255	-.248	-2.222	**.029**	-34.770	-1.909					
	**REMD**	2.758	.953	.329	2.895	**.005**	.862	4.655					
	**RMSSD**	-12.436	5.853	-.225	-2.125	**.037**	-24.085	-.787					

Table S4. Hierarchical regression analysis for subjective extinction recall (sERI). Addition of RMSSD significantly increased the proportion of variance explained by the model. Note that smaller sERI denotes better extinction recall. REMD: REM density; RMSSD: Root mean square of successive differences.

								ANOVA		
**Predictors**	**B**	**SE**	**β**	**t**	**p**	**95% CI**		**Adj. R^2**	**F**	**p**
**Sex**	-.604	.325	-.236	-1.856	.068	-1.255	.046	.141	3.702	**.009**
**%REM**	-.050	.023	-.271	-2.190	**.032**	-.096	-.004			
**REMD**	.139	.051	.362	2.755	**.008**	.038	.241			
**REML**	-.009	.003	-.359	-2.933	**.005**	-.016	-.003			

Table S5. Linear regression analysis for physiological extinction recall (ERI). Non-contributory predictors included in the original model (see the manuscript) are removed. Note that smaller ERI denotes better extinction recall. %REM: Proportion of REM sleep to the total sleep time; REMD: REM density; REML: REM latency.

								ANOVA		
**Predictors**	**B**	**SE**	**β**	**t**	**p**	**95% CI**		**Adj. R^2**	**F**	**p**
**REMD**	2.363	.876	.268	2.698	**.008**	.624	4.103	.062	7.277	**.008**

Table S6. Linear regression analysis for subjective extinction recall (sERI). Non-contributory predictors included in the original model (see the manuscript) are removed. Note that smaller sERI denotes better extinction recall. REMD: REM density.

								ANOVA		
**Predictors**	**B**	**SE**	**β**	**t**	**p**	**95% CI**		**Adj. R^2**	**F**	**p**
**Sex**	-.559	.324	-.224	-1.726	.090	-1.208	.090	.179	3.177	**.010**
**%REM**	.158	.052	.420	3.063	**.003**	.055	.261			
**REMD**	-.051	.024	-.273	-2.115	**.039**	-.099	-.003			
**REML**	-.010	.003	-.392	-3.024	**.004**	-.017	-.003			
**HF[ms^2]**	-.413	.198	-.356	-2.092	**.041**	-.809	-.017			
**HF[ms^2]×Sex**	.630	.285	.389	2.211	**.031**	.059	1.202			

Table S7. Final model in the hierarchical regression analysis for physiological extinction recall (ERI), after non-contributory predictors included in the original model (see the manuscript) are removed. Note that smaller ERI denotes better extinction recall. %REM: Proportion of REM sleep to the total sleep time; HF[ms^2]: Absolute power of high frequency heart rate variability; REMD: REM density; REML: REM latency.

								ANOVA		
**Predictors**	**B**	**SE**	**β**	**t**	**p**	**95% CI**		**Adj. R^2**	**F**	**p**
**Medications**	-17.781	8.015	-.241	-2.218	**.029**	-33.727	-1.834	.140	5.568	**.002**
**REMD**	2.722	.902	.324	3.019	**.003**	.928	4.516			
**HF[ms^2]**	-6.619	2.877	-.240	-2.301	**.024**	-12.343	-.895			

Table S8. Final model in the hierarchical regression analysis for subjective extinction recall (sERI), after non-contributory predictors included in the original model (see the manuscript) are removed. Note that smaller sERI denotes better extinction recall. %REM: Proportion of REM sleep to the total sleep time; HF[ms^2]: Absolute power of high frequency heart rate variability; REMD: REM density.

Reliability of Heart Rate Variability Measures Across Baseline and Consolidation Nights

To examine the stability of vagally mediated heart rate variability across baseline and consolidation nights, we calculated the intraclass correlation coefficients (ICC) for both HF[ms^2] and RMSSD. Heart rate variability indexes from the baseline night was reported in a previous study (Daffre et al. 2023), which calculated the HRV metrics differently from the current study. In the previous study, time-weighted averages (in contrast to the simple averages in the current study) were used for all indexes, and frequency domain metrics were calculated using Fourier transformation (in contrast to the autoregressive method used in the current study). Despite these methodological differences, both indexes showed high reliability across the two nights:

	**ICC**	**95% Confidence Interval**	
**HF-HRV**	0.92	.864	.948
**RMSSD**	0.90	.835	.936

*HF-HRV: High-frequency heart rate variability; ICC: Intraclass correlation coefficient.

Association of Heart Rate Variability With Demographic and Clinical Variables

HF[ms^2] was not associated with age ($F(1,89)=1.137$, $p=0.289$), sex ($F(1,89)=0.199$, $p=0.656$), PTSD diagnosis ($F(1,89)=.264$, $p=0.608$), depressive symptom severity as measured by Quick Inventory of Depressive Symptomatology (QIDS; $F(1,89)=0.795$, $p=0.375$) or the severity of posttraumatic stress symptoms as measured by Clinician-Administered PTSD Scale for DSM-5 (CAPS-5; $F(1,89)=0.444$, $p=0.507$). Medication use (benzodiazepines or antidepressants) was associated with lower HF[ms^2] ($F(1,89)=5.123$, $p=0.026$).

Daffre C, Oliver KI, Nazareno JRS, Mader T, Seo J, Dominguez JP, Gannon K, Lasko NB, Orr SP and Pace-Schott EF (2023) Rapid eye movement sleep parasympathetic activity predicts wake hyperarousal symptoms following a traumatic event. *J Sleep Res* **32**(1)**,** e13685. https://doi.org/10.1111/jsr.13685.
